# Normalization of technology for social contact in a Norwegian care facility during COVID-19

**DOI:** 10.1186/s12913-022-08618-7

**Published:** 2022-10-14

**Authors:** Abeer Badawy, Mads Solberg, Aud Uhlen Obstfelder, Rigmor Einang Alnes

**Affiliations:** 1 Department of Health Sciences, Faculty of Medicine and Health Sciences, Norwegian University of Science and Technology, Larsgårdsvegen 2, 6009 Ålesund, Norway; 2 Center for Care Research, Department of Health Sciences, Faculty of Medicine and Health Sciences, Norwegian University of Science and Technology, Teknologivegen 22, 2815 Gjøvik, Norway

**Keywords:** Interactive technology, Social contact, Normalization process theory, Implementation, Case study, Care facilities, Older people

## Abstract

**Background:**

The COVID-19 pandemic has seen unprecedented growth in the use of interactive technologies in care facilities for social contact between residents and their close contacts due to the need for social distancing. As the pandemic is transitioning into a new phase, there is a need to critically examine the new practices associated with technology usage.

**Objective:**

Our analysis is based on a case study of how a care facility in western Norway adopted a novel technology called KOMP. We empirically investigate the stability of practices with KOMP for maintaining social communication between residents and their relatives and consider whether these practices are likely to last beyond the pandemic. We draw on normalization process theory (NPT) to interpret our findings and critically examine how stable embedding of new technologies for social communication occurs under extraordinary circumstances.

**Methods:**

We conducted a case study based on participant observation and interviews, and the data were analyzed through inductive thematic analysis. Participants are health care professionals from a public care facility in western Norway.

**Results:**

Four major themes emerged from the data. The first revolved around the pressing need for communications between residents and relatives with a suitable tool. Second, staff showed engagement through motivation to learn and adapt the technology in their practices. A third theme centered on how staff and the organization could work effectively to embed KOMP in daily practice. Our fourth theme suggested that the professionals continuously assessed their own use of the technology.

**Conclusion:**

From the perspective of NPT, practices with KOMP have been partially embedded by developing a shared understanding, engaging through cognitive participation, working collectively with staff and the organization, and reflexively monitoring the benefits of using KOMP. However, staff engagement with the technology was continuously threatened by factors related to diverging staff preferences, the burden of facilitating KOMP for residents with impaired cognitive and physical abilities, issues of privacy and ethics, and the technical skills of the residents’ relatives. Our analysis suggests that caring practices via KOMP have become relatively stable despite barriers to engagement and are therefore likely to persist beyond the pandemic.

## Introduction

During the COVID-19 pandemic, elderly residents in Norwegian care facilities have experienced severe restrictions on visitations from their relatives, which can negatively affect their social relationships and wellbeing [1–4]. Maintaining a social network and relationships with family and friends are widely considered to benefit the quality of life of older adults [5, 6]. Today, many technologies enable the maintenance of relationships at a distance, such as telephones, messaging services, and video calls [3, 7, 8]. In an affluent welfare state such as Norway, residents and staff in many public care facilities have access to a diverse group of digital tools for supporting communication and maintaining social contact [9, 10].

More recently, a tablet-like device known as KOMP (a name derived from Norwegian *kompis*, meaning ‘buddy’) was introduced to a limited extent in some care facilities in western Norway. Known as “The one-button computer connecting generations”, KOMP was adopted by care facilities so that residents can maintain contact with their families during the pandemic. An important question is therefore whether the use of KOMP has become a stable routine for digital communication in these contexts. By stability, we here mean the regular facilitation of digital communication through KOMP by health care staff amidst other everyday caring tasks, promoting long-term use.

The use of such a technology at this moment is not unique to the Norwegian context, as health care organizations worldwide have moved rapidly to introduce technologies for social contact to support quality of life under critical conditions during the pandemic, instilling new practices for care and affecting the nature of the care environment [11, 12]. Consequently, health care workers and organizations have had to modify their work regimes [13, 14]. To achieve the goal of productively using a technology for social contact with elderly users, it is essential to empirically understand causes of stability and instability in the long term [15]. Research on the sustainable use of technologies for social contact among this user group, however, is scarce [15].

In a previous publication, we documented aspects of this ad hoc use of KOMP during the COVID-19 pandemic, revealing multiple aspects of new and modified routines associated with KOMP usage [16]. In this article, we examine a different set of issues and practices, namely the normalization of KOMP adoption in care facilities, considering the potential stability of these practices beyond the pandemic. To understand the extent and consequences of normalization, we adopt a theoretical framework known as Normalization Process Theory, to discuss how staff engage with KOMP as part of everyday care within the facility.

Several theoretical frameworks and models attempt to describe how new technologies are implemented in health care practice [17, 18]. Examples include the quality implementation framework; the active implementation framework; the conceptual model; diffusion of innovations theory and the implementation effectiveness model [17–19]. These frameworks, theories and models have different assumptions, aims, and characteristics and entail different commitments for the researcher who uses them [17]. For instance, the active implementation framework and the conceptual model aim to explain, predict, or interpret implementation outcomes. Other classic theories account for mechanisms of change and how this occurs in implementation. Evaluation frameworks, on the other hand, determine implementation success [17].

Normalization process theory (NPT), however, is a theory of implementation centered on identifying the situated actions that workers take to ensure the routine embedding of new technologies and sustained embedded practices, i.e., “integration” [17, 19, 20]. NPT identifies four core constructs (coherence, cognitive participation, collective action, and reflection) that represent generative mechanisms for social action and the work that professionals do to routinely make use of new technology in health care. The framework offers a processual account of new technology in the workplace and why some work practices become normalized within organizations, while others do not [20, 21]. For new technologies to accomplish their goals, we need to be reflexive about how they become “normalized” in work practices [11]. Through reflexivity about how normalization occurs in healthcare, it is possible to better understand, recognize and disclose practical implementation problems with specific technologies [22].

Based on experiences accumulated from recent efforts to introduce KOMP in the context of Norwegian care facilities, we empirically investigate the stability of practices with KOMP for maintaining social communication between residents and their relatives and consider whether these practices are likely to last beyond the pandemic. Practices associated with KOMP usage will be examined in light of NPT to answer whether these practices stabilized during the pandemic.

## Methods

### Research design

This case study centers on a public short-term care facility in western Norway. Specifically, we follow the work of health care professionals in one organization as they grapple with interactive technology for social contact during COVID-19, accommodating this novel tool as part of their daily practice under exceptional circumstances. Drawing on NPT as an analytical framework, we then consider how KOMP was accommodated and stabilized in everyday caring practice. The case was delineated by the organizational and physical boundaries of the care facility in question, aiming toward analytical and conceptual generalization, not statistical representativity [24], p. 20. Our particular case made it possible to use three different methods, including a focus group, interviews with individuals, and participant observation, to gain an in-depth understanding of how KOMP was used in this real-world context [23, 24]. First, we carried out a focus group interview in the care facility with a moderated dialog to gain initial insights about the experiences, views, and beliefs of health care professionals about KOMP and related practices [25]. Then, individual interviews were conducted to generate in-depth responses about the informants’ experiences, perceptions, and feelings about KOMP [26]. Finally, we performed participant observation to better understand how health care professionals engaged with the technology in a naturalistic context [24, 27].

### The case

The care facility had two wards for short-term stays ranging from two to eight weeks in duration. However, due to long waiting lists for long-term care, it was not uncommon that residents had to prolong their stays for up to two years. The two wards had a total of 31 residents between 85 and 100 years of age. It was estimated that approximately 80% of care facility residents in Norway live with some kind of cognitive impairment according to Mjørud et al. [28]. There were six to seven health care professionals on the morning shift (i.e., registered nurses, assistant nurses, an activity manager, and sometimes two nursing students) and two to three health care professionals on the evening shift. Like many other care facilities in Norway, this facility had some experience using interactive technologies, including smartphones and iPads, with residents so they could maintain social contact with their relatives. Before the pandemic, the facility had also begun experimenting with KOMP, but its use was limited to a couple of residents at first. However, as we began collecting data during the pandemic in the fall of 2020, the number of residents who used KOMP increased to eleven.

### KOMP

**Fig. 1:**
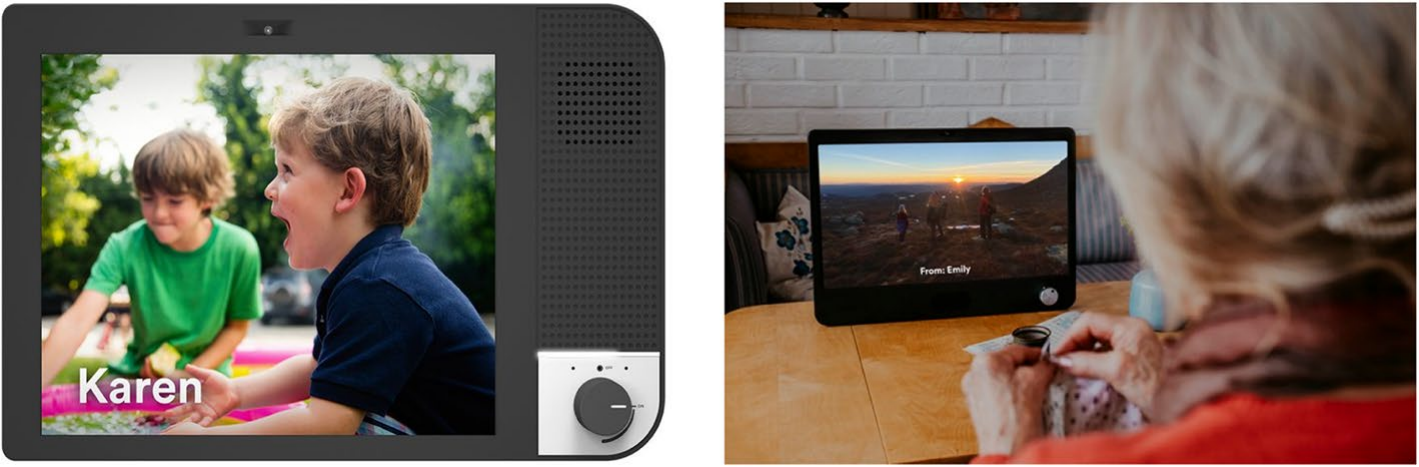
KOMP from No Isolation (©Photographer Estera K. Johnsrud)

As illustrated in Fig. 1, KOMP is a technology for social contact designed so those elderly individuals can interact with their families and friends by sharing pictures, exchanging text messages, and entertaining video calls. KOMP comes equipped with a 17-inch screen, an eight-megapixel camera, a microphone and speaker, Wi-Fi technology, and an easy-to-grip adjustment knob. The large screen is ideal for users with poor eyesight, and the interface is not based on touch response to avoid problems with capacitive sensing. KOMP also amplifies sound to be suitable for users who have hearing difficulties [29].

### Sampling

The first and last authors began the recruitment process in August 2020 by contacting the person in charge of assistive living technology at the municipality to gain information about care facilities that had experiences using KOMP. Based on this information, emails were sent to sixteen care facilities in the same county. Emails included a study description, an invitation to participate with a consent form, and the contact information for both the first and last author. One care facility agreed to contribute by allowing the research team to interview staff (after voluntary consent) and carry out participant observation within their organization. In addition, five health care professionals from this particular care facility, including two managers of different wards, two registered nurses, and an assistant nurse, agreed to attend a focus group interview. In November 2020, observations were conducted for six days during the morning shift. Ten health care professionals, including five registered nurses, two assistant nurses, two care facility physicians, and an activity manager, agreed to participate and share their experiences through individual interviews. They also allowed the main author to conduct observations of their work. In sum, the study included fifteen health care professionals, thirteen women, and two men. One informant attended both the focus group and an individual interview.

### Data collection

Data collection began in September and lasted throughout November 2020. The focus group was carried out in person at the meeting room in the care facility. Participant observations were performed by the first author in two wards in the short-term care facility in November 2020 for three days in each ward, mainly during the morning shift. The first author’s role as a researcher was disclosed to all participants [27]. These observations were documented through field notes. The eleven individual interviews were conducted separately in the nursing room.

A semi structured interview guide based on open-ended questions was used for both the focus group interview and the eleven individual interviews. The focus group interview was carried out by the first and last authors. The first author hosted the interview, while the last author took notes and documented the meeting content in a session lasting one hour. Individual interviews with health care professionals were then conducted by the first author, lasting between eight and twenty-four minutes (average: fourteen minutes). All interviews were digitally recorded and transcribed verbatim. Descriptive fieldnotes from observational sessions were transcribed in Norwegian. The first author transcribed the interviews and observational field notes.

### Data management and ethics

The Regional Ethics Committee declared the study to fall outside the jurisdiction of the Act on Medical and Health Research, and the study was assessed and recommended by the Data Protection Official for Research at the Norwegian Centre for Research Data (ref. 108323). All personal data were collected based on informed consent. Notably, participation was voluntary, and the research subjects could withdraw their consent at any point. All data in the study have been anonymized, and the research was carried out in accordance with the Helsinki Declaration.

### Data analysis

To identify patterns of meaning concerning the use of KOMP and its role in caring practice at the facility, we performed a systematic, thematic analysis with an inductive approach, inspired by Braun and Clarke [30]. In the first phase of analysis, we thoroughly familiarized ourselves with the interview transcripts and field notes, determining the most interesting and relevant units of meaning regarding the use of interactive technology for maintaining social communication between residents and their relatives. Second, we generated an initial list of salient codes using NVivo 12 (version 1.3) based on systematic inquiry across the whole dataset. From this process, several relevant code extracts emerged, resulting in the initial codes.

**Fig. 2:**
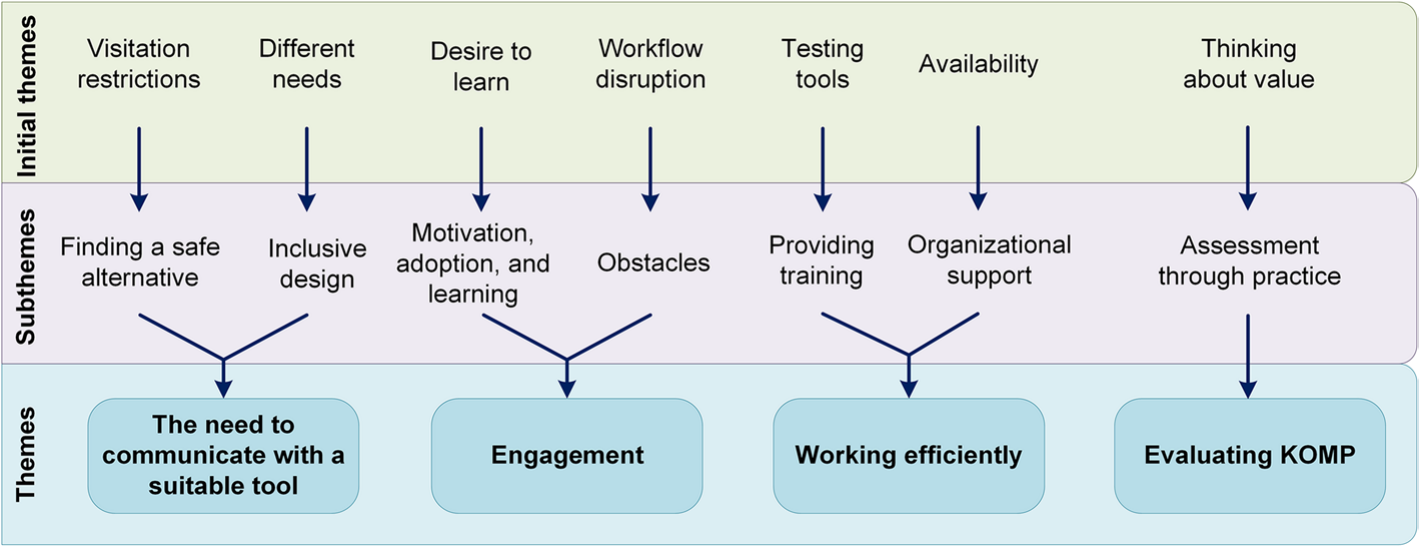
Illustration of the inductive process from the identification of the initial themes to the identification of the four overarching themes

We then organized and mapped this information in the software MindManager, a ‘virtual whiteboard’. To further condense these data and identify broader thematic elements, we regrouped initial codes with a high degree of similarity under a new subtheme, generating seven subthemes. Using MindManager, we were able to visualize the interrelations between codes. Based on interconnections between seven subthemes, this assortment, in turn, coalesced into four overarching themes. Table 1 and Fig. 2 illustrate this inductive process, from the selection of code extracts, via initial codes and subthemes, to the identification of the four final themes. Below, we further unpack this coding scheme.

**Table 1:** Examples from the inductive process of code extraction, initial codes, subthemes, and themes

Code extract	Initial code	Subtheme	Theme
“Residents had flexible visiting hours and daily activities before COVID-19, but they have been very restricted”	Visitation restrictions	Finding a safe alternative	The need to communicate with a suitable tool
“I think that some relatives may experience more safety when using KOMP because they can contact their loved ones frequently”	Safe and frequent communication		
“I think that it is very positive that KOMP has a large screen, particularly for residents with poor vision”	Different needs	Inclusive design	
“We think that more staff want to learn about technology”	Desire to learn	Motivation, adoption, and learning	Engagement
“We struggle with some older relatives when they are unable to download the KOMP application on their phones to send pictures”	Workflow disruption	Obstacles	
“We have four KOMPs in each ward—the more, the better”	Availability	Organizational support	Working efficiently
“We have a daily whiteboard meeting in each ward, where we plan out the daily tasks and organize digital tool testing”	Testing tools	Providing training	
“KOMP stimulates the residents in many ways so that they never get bored and takes over the social engagement process in a way that is better than what we can do”	KOMP as a stimulus	Assessment through practice	Evaluating KOMP
“We must think that it makes sense for us to use KOMP; it should be a useful tool”	Thinking about value

## Results

Our thematic analysis revealed four central themes described below that represent the working practices within the care facility when KOMP was used.

### The need to communicate with a suitable tool

Staff understood the necessity of communicating digitally due to drastic measures to enforce social distancing between residents and their relatives, and they actively highlighted the differences between KOMP and iPads or smartphones after KOMP was available in the care facility.

#### Finding a safe alternative

As the pandemic progressed, health care professionals had to deal with a dramatic change in everyday social life at the facility, particularly with respect to social contact between residents and families and other social activities due to new regulations on visitations to the facility. In the words of one nurse, “Residents had flexible visiting hours and daily activities before COVID-19, but they have been very restricted”. Staff also noticed that these limitations on social life had a negative impact on the residents they cared for, and they soon realized that one of the only ways of maintaining social connections between the frail residents and their relatives in compliance with the need for social distancing was by facilitating digital communications. They addressed the increased need to use KOMP when visits were not allowed. A consensus emerged among staff that KOMP offered an alternative and safe way to keep the residents socially connected and engaged. Staff reasoned that residents’ relatives may have had the same impression about the use value of KOMP under these conditions. As one nurse mentioned, “I think that some relatives may experience more safety when using KOMP because they can contact their loved ones frequently”. Staff therefore strived to embed KOMP as a normal part of their daily routines. They would, for instance, offer KOMP to newly admitted residents and make sure that the device was turned on when each resident woke up in the morning so that it could display photos and receive video calls.

#### Inclusive design

Based on experiences from an intensified use of technology during the pandemic, staff also highlighted differences between KOMP and iPads or smartphones in our interviews. In particular, the many features of KOMP accentuated various affordances of different devices they had used to facilitate social contact. As one nurse explained, they had substituted iPads for KOMPs with some residents since KOMP was suitable for residents with quite different needs. Another issue favoring the use of KOMP was the frequent log-in problems staff had experienced with iPads and smartphones. Furthermore, KOMP’s simple user interface was seen as convenient and appropriate for users with cognitive and physical impairments.

Using smartphones and iPads, for instance, often entailed problems with log-in (usernames and passwords) and necessitated dedicated apps for calls, messaging, and photo sharing with relatives. Staff also remarked how making iPads and smartphones work adequately to facilitate social interactions between residents and relatives outside the care facility required assistance from either a caregiver or another family member. Those residents who required less assistance from a caregiver also used KOMP more frequently. As explained by one nurse, “When a call goes directly from relatives to the resident without the intervention of health care professionals, video calling occurs more frequently”. Staff considered ease of use for older people to be a significant criterion when deciding which digital solution, they should embed as part of their daily routine. Notably, some of the challenges reported by the health care professionals pertained to seemingly mundane hassles, such as lack of internet availability due to poor coverage in the building.

Staff also reported that elderly users sometimes had dry skin on their fingertips, which made it difficult for them to control smart devices based on a touch interface. Conversely, KOMP usage does not rely on a touch interface. Instead, it is a nonportable device with a large screen and an easy-to-grip adjustment knob. The knob is used to switch the KOMP on and off and to adjust the sound volume. Participants in our study also noted that KOMP had a capable loudspeaker, suitable for those with hearing impairments, and that the large 17-inch screen was suitable for those with poor eyesight.

### Engagement

The health care professionals in the study declared that they were motivated to learn about KOMP and adopt the new technology. However, they also identified potential barriers against engagement and use by different actors in the care facility.

#### Motivation, adoption, and learning

We observed that both managers and caregivers in the facility were inclined toward using KOMP with most of the residents whenever possible, and according to the cognitive and physical abilities of each resident. An issue that was first mentioned through interviews, but later confirmed via observations in the care facility, was the role of health care managers in motiving staff by encouraging them to try KOMP in their work and learn how to apply it in activities with eligible residents. The care facility manager presented the following folk theory about how technology diffused within their organization: “Adopting a new technology requires motivated health professionals who desire to change and like to try new technology. This is how change has been done since technology started”. Another interlocutor noticed that widespread use of a new technology necessitated that staff have an interest in changing how they think and that each new technology deserves a fair trial. In the words of one nurse, “We think that more staff want to learn about technology”.

Staff highlighted another facet of working productively with KOMP, namely, the need to motivate and engage the residents themselves, and their families. An event that was observed in a nursing room, illustrates this involvement process. In this case, a nurse had introduced KOMP to a recently admitted resident by presenting the device as a means of communication between the resident and his relatives. She instructed the resident about how he could turn on the KOMP himself, and how he could obtain assistance from staff when this was needed. The nurse then called the resident’s family, described KOMP’s functionality, and demonstrated how they could log in at the KOMP website to communicate via the device. She also showed how they could add other family members to the group. Finally, the nurse gave the family a chance to reflect on its use and ask questions. This example shows how the nurse was motivated to encourage the resident and his relatives to utilize KOMP as part of their daily social routine.

#### Obstacles

Although the stakeholders in the facility were actively engaged in adopting KOMP through everyday practices, we also identified factors that could influence their engagement, with possible implications for the long-term trajectory of KOMP usage. These factors pertained to staff preferences, challenges with residents who had cognitive and physical impairments, issues of privacy and ethical dilemmas when using KOMP, and the technical abilities of older relatives.

Staff reported to us that they had quite variable experiences when interacting with technologies such as KOMP. They also had different attitudes, preferences, and skills, which led them to respond quite differently in diverse situations involving the technology. For instance, some workers had voiced their skepticism quite loudly before trying out KOMP in their work practices, and accordingly, they did not have high expectations about its use value. Since most residents could not even use smartphones, they reasoned, how would they receive any benefit from KOMP or even understand its purpose?

One issue that was repeatedly emphasized by the health care staff in the facility was the need for a certain level of cognitive and physical functionality among residents for them to be able to meaningfully socialize with others using KOMP. Unsurprisingly, staff saw considerable variations in the motivations of residents toward its use. One persisting challenge was getting cognitively impaired residents involved in social activities using the device. These residents were often confused and did not understand the purpose of KOMP or why they should take an interest in it. In cases where residents had advanced dementia, they also struggled to recognize their interlocutors on the device, such as having problems connecting specific voices to the source on the screen, potentially causing distress, confusion, and anxiety. While familiar with television and telephones, elderly residents with dementia were not used to multimodal, two-way communication technologies that combined voice and live imagery. As relatives could “dial in” using the app whenever KOMP was turned on, staff-related stories about patients who were startled when relatives abruptly began talking to them through the screen. Based on these experiences, staff had to spend more time and effort helping those users who needed caregivers’ assistance with the device at the potential expense of other residents.

In the care facility, we observed a salient phenomenon related to this issue, namely, that when residents with dementia used KOMP for video communications, the door to their room would be kept open. Residents without dementia, on the other hand, would usually take video calls more privately, with their doors closed. Caregivers explained this peculiar arrangement as a consequence of their need to listen for any incoming calls so that they could quickly facilitate conversations for residents if needed. On occasion, some residents also became annoyed during video calls and thus required immediate assistance. By keeping the doors open, staff could be attentive and intervene to address communication problems when needed. This was not a case of staff “listening in”, but it still suggests that using KOMP as a tool for personalized care with cognitively impaired residents raised dilemmas related to privacy and ethics that health care workers had to address on the spot in specific situations and had to work around.

In interviews, staff also reported that they sometimes struggled with making KOMP work for interacting with residents’ older relatives, who found downloading the app to make calls or to send pictures and text messages difficult. As relayed by one nurse, “We struggle with some older relatives when they are unable to download the KOMP application on their phones to send pictures”. They recognized that new technologies were difficult to implement and that age was an important factor in terms of becoming familiar with its use. As such, KOMP was not considered a panacea to the problem of social contact: some residents were over 90 years old and probably had children well over the age of sixty who lacked the technical knowledge needed to deal efficiently with smartphones and mobile apps to call or send pictures.

### Working efficiently

To facilitate the use of KOMP in everyday life, there was a demand for training to use KOMP, as well as organizational support.

#### Providing training

As part of their efforts, the care facility offered training sessions for the staff before they made use of the digital solution with patients. The everyday use of KOMP was planned ahead administratively, with one manager referring to staff meetings as an important arena for coordinating activity throughout the day: “We have a daily whiteboard meeting in each ward where we plan out the daily tasks and organize testing the digital tools”. Observations of morning meetings, where the caring tasks were distributed, revealed that KOMP figured in lively discussions among staff. For instance, staff ensured that all available devices were used by residents, and that all the nurses on the shift had the necessary training to facilitate and troubleshoot their use. If needed, nurses could also request a technology facilitator to help identify, solve problems, and develop best practices for KOMP use.

#### Organizational support

At the organizational level, there was also support for adapting the technology into new routines, with respect to the availability of economic resources, provision of IT infrastructure, and IT-support from a dedicated technology facilitator. In interviews, the professionals singled out the managerial role as central for securing adequate infrastructure and other resources so they could deliver high-quality technology-supported care. As one of the managers noted, implementing a new piece of interactive technology required meticulous planning, as well as close follow-up to ensure that staff used it continuously. The informants also mentioned that facilitation of social activities added an extra task. They also identified a need for additions to the workforce to be able to provide an adequate level of social activity for the residents.

The professionals also emphasized how support from a dedicated technology facilitator was a major asset and helped ensure successful outcomes. Regarding the supply side of the technology, one manager noted that while they now had an adequate number of KOMP units available, more devices would still be advantageous: “We have four KOMPs in each ward—the more, the better”. The rationale was that more units meant they could spend less time switching user accounts between residents. A reliable stock of devices, however, did not solve issues with unstable Wi-Fi connections and other infrastructure essential to maintain reliable use. In the frank words of one nurse, “The major problem is, what is the Wi-Fi-password?” Addressing this frustration, the facility manager emphasized that future iterations of KOMP should come with a dedicated 4G mobile internet connection, which was seen as a significant advantage for residents and staff alike, potentially resolving one major frustration among users.

### Evaluating KOMP

#### Assessment through practice

In both interviews and during participant observation, the participants reflected upon the role that KOMP played in facilitating social communication during the pandemic. While several interactive communication tools were in use before the pandemic, they had not been utilized to their full potential in supporting social interactions between residents and their relatives. During the pandemic, however, staff realized the value and potential impact of such tools. As declared by one manager, “It is quality of life to stay connected with relatives”. Staff also emphasized how they intended to continue assisting residents who benefited from KOMP beyond the pandemic. In their view, KOMP was able to attract and stimulate residents with pictures and video conversations in ways that facilitated social engagement. As one nurse explained, “KOMP stimulates the residents in many ways so that they never get bored and takes over the social engagement process in a way that is better than what we can do”. Staff saw themselves as able to adapt to and accommodate KOMP in their caring practices, making it fit into the hectic schedule of everyday work in the ward, as well as to the cognitive and physical abilities of those they cared for.

## Discussion

Our results have revealed a rich set of practices and reflections by health care practitioners concerning KOMP as an interactive technology, extensively used during an extraordinary situation. During a pandemic characterized by social distancing, it quickly became paramount for caretakers to adopt new technology to facilitate social contact between residents and their relatives. According to our data, health care professionals collectively endeavored to accomplish this goal.

We now ask whether these practices for KOMP usage are becoming stable in the sense that they are undergoing normalization processes [20]. The stability of a practice can be considered an outcome of normalization whereby it becomes a normal part of daily tasks resulting in the continuity of practice over time. To address this issue, we interpret emergent themes from our data through the lens of four core theoretical constructs of NPT. Since this framework focuses on the work of embedding and sustaining practices, we discuss the social processes that may lead to routine embedding and durable integration of KOMP usage in the care facility beyond the pandemic.

In their programmatic outline of the NPT framework, May and Finch [20, p. 4] defined the embedding of practices as “making practices routine elements of everyday life” and the integration of practices as “sustaining embedded practices in their social contexts”. Accordingly, implementation processes are driven by four generative mechanisms: coherence, cognitive participation, collective action, and reflexive monitoring. These mechanisms are affected by factors promoting or inhibiting the routine embedding or “normalization” of practice in its social context [20, 21]. According to our data, situational demands during the pandemic required staff to rapidly mature in their understanding of how KOMP could provide residents with safe communication with their families, who were barred from physical visitation. They quickly developed a shared understanding of the usefulness of the device and its potential for easing the burden of social distancing. Having been forced to act rapidly and enforce social distancing at the start of pandemic in March 2020, there was limited time to develop comprehensive strategies and measures for social visits. We believe that the urgency of pandemic measures and the need to identify workable, adequate solutions to maintain social contact between residents and their relatives promoted routine embedding of KOMP practices. During an exceptional period, there seems to have been a radical shift in the mindset of health care staff about the value of technology for digital communication [16].

According to Mair et al. [31], the shared views and understanding that users have when a new technology is implemented can be conceptualized as “coherence”. Through shared understanding, staff were able to implement and realize a new set of practices to establish safe communication through KOMP and make sense of the differences between these practices and former visitation practices, and its impact on everyday work.

Health care professionals at the facility agreed about the convenience of using KOMP compared to other tools (such as iPads and smartphones), given the capacities of their residents. Compared to other tools, staff perceived KOMP to be an accessible tool. Its use could also be tailored to each resident’s abilities, whether this was vision impairments, hearing impairments, or the problem of capacitive sensing with dry fingertips. They also explicitly compared KOMP with more established digital devices, through a process of “differentiation” [31]. Furthermore, the professionals construed practices involving KOMP as coherent by defining the elements composing the practice and how these elements differed from practices involving other technologies [20]. Gradually, KOMP practices became a stable aspect of everyday work at the facility through a coherent and shared set of perceptions and understandings.

The work of embedding technology in normalization processes is influenced by factors that promote or inhibit the participation of actors [20]. Known as “cognitive participation”, this process entails individuals’ enrollment in and legitimization of new technology-based practices aiming to be integrated into practice [31, 32]. Staff were engaged and motivated when facilitating and embedding KOMP in caring practices through processes of learning and skillful adoption. Our reported observation of the keen attempts by one nurse to facilitate the use of KOMP with the family of one resident illustrates one aspect of cognitive participation. Staff were motivated to involve as many residents as possible, in the use of KOMP. No longer facilitating physical visits for their residents, they now facilitated digital visits using KOMP, despite busy schedules. While the perceived value of safe, digital communications stimulated more staff to engage and participate in the embedding of KOMP, our results do, however, suggest nuances in levels of engagement among staff. Our data suggest that engagement was influenced by several factors, including variations in technical expertise and preferences among staff, the physical and cognitive disabilities of residents, privacy and ethical dilemmas, and the technical skills of older relatives who wanted to communicate via KOMP. These factors posed barriers against staff engagement, affecting what Mair et al. [31] calls “legitimization”. Legitimization refers to actions taken by professionals to validate their participation in the embedding process by harnessing the value of KOMP in their own work. In this context, efficacious and ethically sound practices of care were seen as essential for long-term sustainability. For instance, privacy and ethical issues need to be managed to ensure confidentiality for the residents in the ward when they were conversing with family. But as we highlighted in the data above, the hectic schedules of health care workers also required them to occasionally keep doors open, so that they could monitor interaction through KOMP and intervene when needed. Such barriers could potentially affect the sustainability of practice in the long run. In post-pandemic times, however, the affordances of KOMP that were valued at the time of our study could eventually be eclipsed by the detrimental impact of additional “technostress” on health care professionals [11, 14].

Another component of normalization concerns the collective work achieved by the staff in the organization. “Collective work” is defined by Finch et al. [32] as the work done by individuals and organizations to execute a new practice. The production and reproduction of a practice require that actors collectively invest and commit to it [20]. Both managers and staff invested in the use of KOMP with residents so they could communicate safely with relatives and friends. This meant that work was adapted to accommodate KOMP. A central dimension of collective work is “contextual integration”. Contextual integration concerns the presence of organizational support and the integration of practices within a social context [31]. The availability of organizational support in terms of human resources, IT infrastructure, and economic resources played a prominent role in facilitating KOMP usage. For a technology to be integrated into an organization, there is a need to ensure staff training, maintenance, adaptation, and facilitation of its use in specific contexts. KOMP was developed to help elderly residents maintain social contact in an era of mass digitization; however, as our examples from the care facility suggest, many older people in such settings are frail, with reduced cognitive and physical abilities. The fact that managers made resources available in the care facility and motivated staff to incorporate KOMP into everyday work helped the routine embedding of these practices. But as Jacobsen has recently pointed out regarding the use of assistive technology in the context of Norwegian health care, we need to be more realistic about the use value and limitations of such technologies for the frailest users [33].

Staff’s reflections about KOMP’s benefits and drawbacks revealed a final mechanism involved in its stabilization. NPT refers to this aspect of normalization as “reflexive monitoring”. According to Finch et al. [32], this includes considerations of the user experience and the tangible impacts that result from a new practice. Reflexive monitoring can also involve judgments about the utility and effectiveness of a new practice, with reference to socially patterned and institutionally shared beliefs. Health care professionals developed judgments and assessments about KOMP’s ease of use, its social value during social distancing, problematic issues, the effects of KOMP on their work environment, and how they could adapt to these matters. Assessing the benefits of technology promotes its routine embedding, while the unclear benefits of technology for users and health staff can result in these individuals ignoring or rejecting it [20]. The factors that promote or inhibit staff’s evaluation of the benefits of KOMP are related both to their schedules and to residents’ physical and cognitive capabilities. Staff continuously reflected on their practices with KOMP and evaluated the technology in ways that reshaped and accommodated its use across the contexts of care.

At present, KOMP balances different stakeholder needs, offering a convenient interface compared to other digital communications tools. Depending on future design iterations and the developmental trajectory of the device, it may or may not become obsolete over time. But whatever technologies for social contact will appear in the future, these must satisfice the same constraints and practices in elderly care that KOMP currently does. As such, this case study of normalization work, carried out under extraordinary circumstances, offers critical lessons for future work on technologies of care.

### Strengths and limitations

Despite restrictions during the COVID-19 pandemic, gaining access to the care facility presented an opportunity to contextualize data from interviews with observations from a naturalistic context. By exploring one care facility through both interviews and observation, we could compare what health care professionals said with what they did. Triangulation by drawing on different sources of evidence is a considerable asset for case studies [23].

Conducting such a case study can provide knowledge about the impact of social distancing on social contact between frail elderly and their families and the role of technology to reduce this burden. Additionally, using KOMP as an example of communication technology makes other research on different communication technologies relevant and comparable considering the needs of involved stakeholders.

Our choice of this care facility, which has a tradition of using many different AAL devices in their work, comes with some caveats. Clearly, the participants in our study had considerable experience with digital assistive technologies, which likely influenced their experiences with KOMP. We do not, however, have a reason to suspect that our sample of professionals is atypical for the Norwegian context.

A relatively brief observation period (six days) also presents a limitation on our findings, as a longer period would have given us a broader sample of KOMP-related events to analyze. There were also potential biases from observing only the morning shift at work, as the evening shift might face quite different challenges regarding the use of KOMP, such as fewer staff at work. The challenges posed by the COVID-19 pandemic affected both the size of our sample and the observational period. Among other issues, the facility was understaffed due to sick leaves during this critical period, and we were not able to access other care facilities.

We recognize that interviewing managers and subordinates together in the same focus group can sometimes present challenges due to perceived differences in social status and the potential for social desirability bias when answering questions, particularly about sensitive issues in the workplace. In turn, this can affect the richness and truthfulness of how a group of respondents answers a query. Notably, the topic of this study was not a controversial one, in the context of Norwegian care practices. Furthermore, the focus group discussions were characterized by openness, with both staff and managers contributing equally to the conversation [25]. In addition to a relatively egalitarian work-life culture, Norwegian employees also have strong labour rights that protect them from arbitrary retaliation from employers. While respondent bias due to small group dynamics cannot be ruled out in principle, the research team had no reason to suspect that our informants were self-censoring their opinions. Data from the observations did not reveal any inconsistencies with the views articulated by managers and staff during the focus group interviews.

## Conclusion

Our case analysis reveals a rich set of social processes contributing to the embedding of KOMP as a tool for digital communication. Four aspects of these processes were highlighted first through the crucial need for digital communication with a suitable tool between residents and relatives. Second, staff engaged with the technology and were motivated to learn about how it could be adapted to their practices. The third aspect focused on how staff and the organization worked collectively in an efficient way to routinely embed KOMP in daily life. The fourth showed how the staff reflected on KOMP’s benefits and continuously evaluated its use through the practices. Drawing on the NPT framework, we examined these four aspects with respect to four generative mechanisms: coherence, cognitive participation, collective action, and reflexive monitoring. We suggest that practices involving KOMP become partially embedded within the organization. The sustained use of a technology depends on whether it becomes part of the routines and practices for care. In this case, it is likely that KOMP usage, as a practice for social contact, will outlast the pandemic. However, our data also reveal some potential barriers regarding engagement among staff. Thus, we need more longitudinal research on the long-term influences of these technologies on practices of care.

## Data Availability

The datasets generated and analyzed during the current study are not publicly available to protect participants' confidentiality but are available from the corresponding author on reasonable request.

## Abbreviations

AAL: Active-Assisted Living
COVID-19: Coronavirus Disease 2019
NPT: Normalization Process Theory
NSD: Norsk Senter for Forskningsdata (Norwegian Centre for Research Data)
